# Regulatory network changes between cell lines and their tissues of origin

**DOI:** 10.1186/s12864-017-4111-x

**Published:** 2017-09-12

**Authors:** Camila M. Lopes-Ramos, Joseph N. Paulson, Cho-Yi Chen, Marieke L. Kuijjer, Maud Fagny, John Platig, Abhijeet R. Sonawane, Dawn L. DeMeo, John Quackenbush, Kimberly Glass

**Affiliations:** 1 Department of Biostatistics and Computational Biology, Dana-Farber Cancer Institute, Boston, MA USA; 2 Department of Biostatistics, Harvard T.H. Chan School of Public Health, Boston, MA USA; 3 Channing Division of Network Medicine, Brigham and Women’s Hospital, and Harvard Medical School, Boston, MA USA; 4 Division of Pulmonary and Critical Care Medicine, Brigham and Women’s Hospital, Boston, MA USA; 5 Department of Cancer Biology, Dana-Farber Cancer Institute, Boston, MA 02215 USA

**Keywords:** Regulatory networks, Transcriptome, GTEx, Lymphoblastoid cell lines, Fibroblast cell lines

## Abstract

**Background:**

Cell lines are an indispensable tool in biomedical research and often used as surrogates for tissues. Although there are recognized important cellular and transcriptomic differences between cell lines and tissues, a systematic overview of the differences between the regulatory processes of a cell line and those of its tissue of origin has not been conducted. The RNA-Seq data generated by the GTEx project is the first available data resource in which it is possible to perform a large-scale transcriptional and regulatory network analysis comparing cell lines with their tissues of origin.

**Results:**

We compared 127 paired Epstein-Barr virus transformed lymphoblastoid cell lines (LCLs) and whole blood samples, and 244 paired primary fibroblast cell lines and skin samples. While gene expression analysis confirms that these cell lines carry the expression signatures of their primary tissues, albeit at reduced levels, network analysis indicates that expression changes are the cumulative result of many previously unreported alterations in transcription factor (TF) regulation. More specifically, cell cycle genes are over-expressed in cell lines compared to primary tissues, and this alteration in expression is a result of less repressive TF targeting. We confirmed these regulatory changes for four TFs, including SMAD5, using independent ChIP-seq data from ENCODE.

**Conclusions:**

Our results provide novel insights into the regulatory mechanisms controlling the expression differences between cell lines and tissues. The strong changes in TF regulation that we observe suggest that network changes, in addition to transcriptional levels, should be considered when using cell lines as models for tissues.

**Electronic supplementary material:**

The online version of this article (10.1186/s12864-017-4111-x) contains supplementary material, which is available to authorized users.

## Background

Cell lines are an essential tool in cellular and molecular biology, providing a lasting resource that can match a particular genotype and phenotype in a controllable and reproducible setting. Cell lines have accelerated the investigation of many biological processes, however despite their merits as an experimental system, cell lines do not capture tissue complexity and heterogeneity, mainly because they consist of a single cell type that is adapted to grow in culture and lacks interactions with other cell types, the extracellular matrix, or paracrine signaling [1, 2]. Cellular heterogeneity is present even in seemingly homogenous groups of cells [3]. While it has been previously reported that these and other factors can influence cell line gene expression [4–6], the differences in transcription factor (TF) regulation between cell lines and tissues have not been systematically studied. Regulatory network approaches can help elucidate the regulatory processes associated with the differences in expression observed in cell lines when compared to their tissues of origin*.*

Standard transcriptomic analyses typically focus on studying the regulation and function of one or a few genes, and these approaches fail to characterize the complex cellular processes defined by the collective contribution of signaling pathways and cell-type specific regulators. On the other hand, TF regulatory networks provide an intuitive framework for characterizing the combinatorial regulatory effect of TFs on their target genes. These regulatory networks capture and quantitatively model the processes that drive cellular phenotype, with differences in network structure reflecting changes in regulatory processes. For example, in previous work experimentally interrogating the subnetwork around a few TFs it has been possible to uncover patterns of transcriptional regulation associated with cellular differentiation [7], pluripotency [8], and development [9]. More recently, by integrating different types of genomic data it has been possible to model genome-wide regulatory networks [10] and to identify distinct regulation patterns within different cell types [11] or different disease states [12–14]. Many of these network algorithms rely on a large number of expression samples and, until now, regulatory networks have not been used to elucidate the regulatory process differences between cell lines and their tissues of origin mainly because of the lack of large data sets with paired samples.

The Genotype-Tissue Expression (GTEx) project [15] generated a large multi-subject data set that offers an unprecedented opportunity to understand how well a cell line’s regulatory processes recapitulate those of its tissue of origin. GTEx version 6.0 includes RNA-Seq data for 244 paired primary fibroblast cell lines and skin samples and 127 paired Epstein-Barr virus (EBV) transformed lymphoblastoid cell lines (LCLs) and whole blood samples. Primary fibroblasts are a type of finite cell line widely used as model systems because they are easily isolated and grown in culture, and almost never show genetic alterations in oncogenes or tumor suppressors [16, 17]. LCLs are among the most widely created, archived, and analyzed continuous cell lines which, in contrast to finite cell lines, acquire the ability to proliferate indefinitely. LCLs have been extensively genotyped and sequenced as part of large collaborative projects, such as the International HapMap [18], 1000 Genomes [19], ENCODE [20] and GTEx [15] projects. Despite their widespread use, there has been concern about using LCLs to model primary tissues, with two small-scale studies finding differences in gene expression profiles between LCLs and primary B cells [21, 22]. While these studies found genes differentially expressed in LCLs compared to B cells, for example the over-expression of cell cycle genes, the regulatory mechanisms associated with this differential expression are not known.

Here we performed a detailed investigation of gene expression and gene regulatory networks using two cell line and tissue pairs, LCL-vs-blood and fibroblast-vs-skin, to understand the regulatory networks mediating expression differences between the cell lines and their tissues of origin. Although we find that many pathways are preserved between cell lines and their tissues of origin, some biological processes that help define the function of the primary tissue are enriched for genes expressed at lower levels. In addition, we find that LCLs and fibroblast cell lines exhibit large changes in their patterns of TF regulation. For example, while cell cycle genes are over-expressed in cell lines compared to their tissues of origin, they have an overall decrease in negative regulation by TFs that are known to function as repressors. These findings suggest that changes in network properties are useful for understanding alterations in gene regulation between cell lines and their tissues of origin.

## Results

### Pathways differentially expressed between cell lines and their tissues of origin

The GTEx project collected post mortem biopsies from multiple tissues and created LCLs and fibroblast cell lines. For the analyses described here, we used only data from research subjects for whom primary tissue and matching cell lines were available. Data (version 6.0) were available for 127 paired whole blood samples and LCLs, and for 244 paired full-thickness skin biopsies and primary fibroblast cell lines [15]; 89 subjects have data across all four groups. We did not find any clear separation of samples based on the year of analysis by the GTEx project (Additional file 1). The cell lines and tissues express similar numbers of genes mapped to similar functional categories (protein coding, antisense, pseudogene, lincRNA, and other; Additional file 1). Principal component analysis (PCA) showed that gene expression easily distinguishes the four groups (Additional file 1). The first principal component and the majority of the variability (37%) separated blood and LCLs from the skin and fibroblast samples. The second component (22%) separated tissues from cell lines. This indicates that while the samples separate based on their tissues of origin, there is also a significant separation between cell lines and primary tissues. The separation seen in the PCA remains robust when random samples are selected (Additional file 1).

In order to quantify the variability present within each of these four groups of samples, we analyzed gene expression variability across all cell line and tissue groups and observed wider variability in gene expression within tissue samples compared to cell line samples (Additional file 2). We also used an f-test to evaluate the differences in gene expression variance between the groups. We found a higher percentage of genes with significantly greater variance in blood compared to LCL, and in skin compared to fibroblast (FDR < 0.05, Additional file 2). Also, fibroblast have a higher percentage of genes with greater variance than LCL, and blood have a higher percentage than skin. Although some gene expression variability can be attributed to stochastic processes, and tissue heterogeneity may contribute to these observed differences, expression variability is also strongly mediated by the genomic and epigenomic context. For instance, expression variability can be modulated in a tissue-specific fashion and determined by promoter binding affinity [23].

**Fig. 1:**
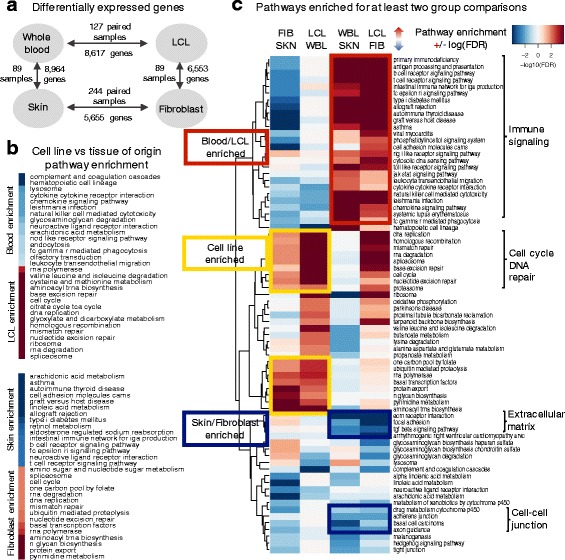
Pathways are differentially expressed between cell lines and their tissues of origin. **a** Number of differentially expressed genes (absolute log_2_ fold change >2 and FDR < 0.05) using voom on paired samples. **b** Results of GSEA reported based on the log_10_(FDR) significance scale, with one group in red and the other one in blue. The 15 pathways most significantly differentially expressed between each cell line and its tissue of origin. **c** Pathways enriched for at least two group comparisons (FDR < 0.05). The pathways differentially expressed between the tissues that are also differentially expressed between the cell lines (preserved pathways) are highlighted in red and blue. Pathways over-expressed in both cell lines compared to their tissues of origin are highlighted in yellow. Rows are ordered by hierarchical clustering of the enrichment significance values, log_10_(FDR). To represent the FDR significance in the heatmap, the color was saturated at 1.1 × 10^−4^. The exact reported FDR can be found in Additional file 2

To explore gene regulation differences between cell lines and their tissues of origin, we sought to identify both differentially expressed genes and the pathways associated with them, followed by an analysis of the drivers of the transcriptional changes through gene regulatory network analysis. We used voom [24] and Gene Set Enrichment Analysis (GSEA) [25] to identify biological pathways that are enriched in genes differentially expressed between cell lines and their tissues of origin. We found 8617 genes (32%) to be differentially expressed between LCL and blood samples (absolute log_2_ fold change >2 and FDR < 0.05) with most of the differentially expressed genes (71%) over-expressed in LCL samples (Fig. 1a, Additional files 3 and 4). For the fibroblast-vs-skin comparison, we identified 5655 differentially expressed genes (21%). In contrast to the LCL-vs-blood comparison, most of the differentially expressed genes (68%) had increased expression in the primary tissue rather than in the cell line.

Using GSEA, with genes ranked by the moderated *t*-statistic from voom, we identified Kyoto Encyclopedia of Genes and Genomes (KEGG) pathways [26] enriched for differentially expressed genes between cell lines and their tissues of origin. Consistent with the separation observed in the PCA, both cell lines exhibit enrichment for pathways with similar biological functions compared to their tissues of origin (Fig. 1b). While immune processes are down-regulated in cell lines, the pathways with positive enrichment are generally associated with cellular growth, and include cell cycle, DNA replication and repair, and transcription processes.

When comparing blood to LCLs, we found that pathways enriched in blood were related to immune system function, including complement and coagulation cascades, hematopoietic cell lineage, chemokine signaling, and natural killer cell mediated cytotoxicity, while the pathways enriched in LCLs were associated with cell growth and death, DNA replication and repair, transcription, and metabolism (FDR < 0.05, Fig. 1b). Similarly, when comparing skin to fibroblasts, the pathways enriched in skin were related to the immune system, metabolism, cell adhesion, and melanogenesis, while the pathways enriched in fibroblasts were associated with cell growth and death, DNA replication and repair, transcription, and protein degradation (FDR < 0.05, Fig. 1b). The significance of all KEGG pathways is listed in Additional file 5.

We also performed differential expression and KEGG pathway enrichment analysis comparing the two tissues and comparing the two cell lines (Fig. 1c). We found a number of immune signaling pathways enriched in blood compared to skin and in LCLs compared to fibroblasts. For example, pathways related to the biological function of B cells (B cell receptor signaling, toll-like receptor signaling, antigen processing and presentation) were enriched in LCL and blood samples when comparing them to fibroblast and skin samples, respectively. However, some immune related pathways, including chemokine signaling and natural killer cell mediated cytotoxicity, were also enriched in LCL and blood samples compared to fibroblast and skin samples, but they were expressed at lower levels in LCL compared to blood samples. As observed previously, these immune signaling pathways are also significantly depleted in fibroblasts compared to skin, which is a tissue with a key role in immunity and associated with many immune cell types [27].

The pathways enriched in fibroblast and skin samples compared to LCL and blood samples, respectively, were associated with biological processes related to maintaining skin structure and organization and included cell-cell junction, extracellular matrix interaction, and transforming growth factor beta (TGF-β) signaling. We find that pathways related to cell cycle and DNA repair are enriched in cell lines compared to their tissues of origin, and more enriched in LCLs compared to fibroblasts.

Overall, we found that the preserved pathways in cell lines are mainly related to the cell type specific functions (B cells or fibroblasts) rather than tissue-enriched functions. Further, many of the genes in pathways that help define the function of the tissue are expressed at a lower level in cell lines relative to their tissues of origin.

### Cell line and tissue-specific gene regulatory networks

Understanding the structure of gene regulation in cell lines compared to their tissues of origin has the potential to help interpret the differential expression results and to reveal important regulatory differences. PANDA (Passing Attributes between Networks for Data Assimilation) is an approach that integrates multiple types of genomic data to infer the network of interactions between TFs and their target genes [28]. In contrast to other network reconstruction approaches, PANDA searches for consistency across multiple sources of information in order to build a holistic regulatory model. The core of the PANDA algorithm is a message passing approach in which regulatory processes are modeled as a communication process between “transmitters” (TFs) and “receivers” (target genes). For communication to occur, both transmitters and receivers play an active role: TFs are responsible for regulating genes and the target genes must be available to be regulated. PANDA starts with a TF/target gene prior regulatory network consisting of potential routes for communication, which is built by mapping TFs motifs to the genome. PANDA integrates this prior network with protein-protein interaction (PPI) and gene expression data, using it to model TF cooperativity and gene co-expression, respectively. Based on this information, it then iteratively estimates the most likely routes of communication through the regulatory network.

We used PANDA to estimate gene regulatory networks in LCL, blood, fibroblast, and skin (Additional file 6). For each network, we began with the same TF/target gene prior regulatory network and PPI prior network, but used “tissue”-specific gene expression data. This resulted in four gene regulatory networks where each edge connects a TF to a target gene, and the associated edge weight indicates the strength of the inferred regulatory relationship in that “tissue”. These networks can inform us about the genome-wide regulation of the cell lines and tissues analyzed as we compare 652 TFs, 27,175 target genes, and more than 17 million edges between them.

We used bootstrapping to select random sets of RNA-Seq expression data to estimate the robustness of these network models, generating 100 random networks for each of the cell line or tissue groups (Additional file 6). We observed a high level of consistency across the bootstrapped networks; the average weight of the edges across these networks is highly similar to the weights of the edges in the network estimated using all samples (Pearson correlation ≥0.98).

**Fig. 2:**
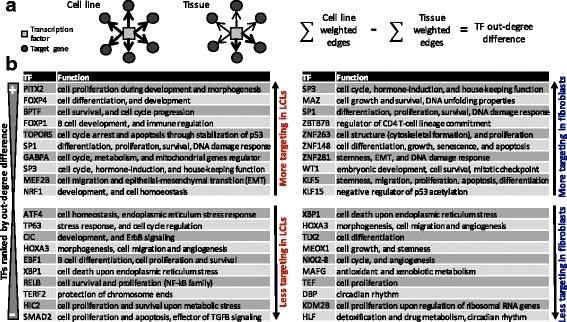
Transcription factors differentially-targeting genes in cell lines and their tissues of origin. **a** Illustration of the TF out-degree difference between each cell line and its tissue of origin. Positive values indicate higher targeting in cell lines, and negative values indicate higher targeting in tissues. **b** Function of the TFs with the largest difference in out-degree comparing LCL-vs-blood; and fibroblast-vs-skin regulatory networks. The complete table with references and differential expression results is shown in Additional file 8

For each TF we computed the difference between the “out-degree” (sum of edge weights from that TF) in the cell line network and the corresponding tissue of origin network (Fig. 2a); these values are also highly robust across our bootstrapped networks (Additional file 6). We ranked TFs by their absolute difference in out-degree (differential targeting, Additional file 7) and found that TFs with the largest differential targeting were involved in cellular responses to stress and DNA damage and in the control of cellular growth (Fig. 2b, Additional file 8). For both the LCL-vs-blood and fibroblast-vs-skin comparisons, many of the top differentially-targeting TFs, such as tumor protein p63 (TP63), TOP1 binding arginine/serine rich protein (TOPORS), and Kruppel like factor 15 (KLF15), belong to the p53 family or interact with p53 and are important mediators of DNA damage response regulating cell cycle arrest, DNA repair and apoptosis [29–32].

We found Sp1 transcription factor (SP1) and Sp3 transcription factor (SP3) had increased targeting in cell lines in both the LCL-vs-blood and fibroblast-vs-skin network comparisons. These TFs have more than 12,000 binding sites in the human genome and are involved in essential cellular processes, including proliferation, differentiation, and DNA damage response [33, 34]. It is important to note that SP1 and SP3 are not differentially expressed between cell line and tissue samples (Additional file 8). However, network comparisons captured the regulatory “rewiring” of these TFs and their target genes, revealing potential differences in targeting even in cases where the TFs themselves were not differentially expressed. Thus, our network models suggest that TFs alter their patterns of regulation in cell lines, either through changing their expression or altering the genes they target (Additional file 7).

### Cell cycle pathway genes are less strongly targeted by TFs in cell lines

We tested whether or not changes in inferred TF targeting preferentially affected genes belonging to specific biological pathways. Similar to the TF’s out-degree, we calculated each gene’s “in-degree” as the sum of edge weights connected to a gene, which represents how strongly targeted each gene is by the complete set of TFs. Again, we find that these values are highly robust across the bootstrapped networks (Additional file 6). We compared the in-degree differences between cell lines and tissues for genes of a specific pathway against all other genes using an unpaired *t*-test (Additional file 9). For the pathways over-expressed in the cell lines, such as cell cycle, DNA repair, and DNA replication, we found a marked reduction of targeting in cell lines compared to their tissues of origin (Additional file 10).

**Fig. 3:**
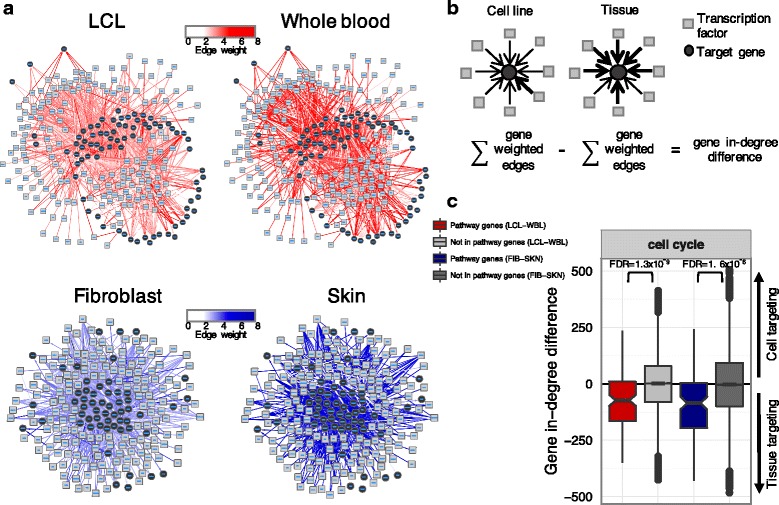
Cell cycle pathway genes are less strongly targeted by TFs in cell lines. **a** Group-specific gene regulatory networks were generated using PANDA. The illustrations represent subnetworks of the 1000 edges with the highest edge weight difference between a cell line and its tissue of origin around the cell cycle genes. The color indicates the edge weight strength between the TF and target gene (the edges shown have a weight greater than 2 in at least one network). **b** Illustration of the gene in-degree difference between each cell line and its tissue of origin. Positive values indicate higher targeting in cell lines, and negative values indicate higher targeting in tissues. **c** Boxplot of the gene in-degree differences for the genes in the KEGG cell cycle pathway and for genes not in this pathway (significance measured using a *t*-test). Reduction of gene in-degree difference indicates that the genes in the cell cycle pathway are less strongly targeted by TFs in the cell line compared to its tissue of origin

**Fig. 4:**
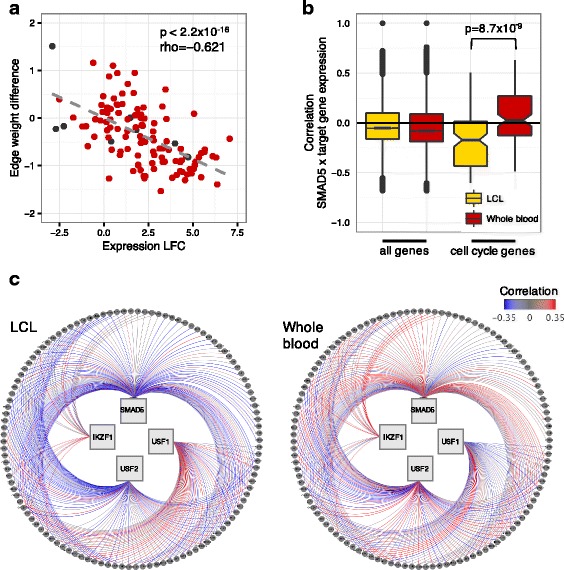
SMAD5 is differentially regulating cell cycle pathway genes. **a** Spearman correlation between the log_2_ fold change in gene expression (LCL-blood difference) of KEGG cell cycle pathway genes and the differential targeting they receive by the TF SMAD5. Red: evidence of SMAD5 ChIP-Seq binding in the promoter of the gene, black: no evidence of SMAD5 binding. The negative correlation observed indicates the cell cycle genes are more highly expressed but less targeted by SMAD5 in LCL compared to blood. **b** Boxplot of Spearman correlation coefficients between SMAD5 expression levels and expression levels of all genes, and between SMAD5 expression levels and the expression levels of cell cycle genes with SMAD5 ChIP-Seq binding evidence for LCL and blood samples. Difference in magnitude was tested using a Wilcoxon rank-sum test LCL-vs- blood comparison. **c** Visualization of the correlation between TF and cell cycle gene expression for interactions that have ChIP-Seq binding evidence. More positively-correlated associations are shown in red, more negatively correlated are blue, and correlations near zero are gray

To better understand these differences, we explored the network around 121 genes in the KEGG cell cycle pathway (Fig. 3, cell cycle gene names listed in Additional file 11). When comparing the log_2_ fold change of the expression levels of these genes with their edge weight differences, we found a negative correlation for many TFs. The TFs with the highest negative correlation include SMAD family member 5 (SMAD5) (Fig. 4a), E2F transcription factor 8 (E2F8), zinc finger and BTB domain containing 14 (ZBTB14), ETS variant 5 (ETV5), helicase like transcription factor (HLTF), upstream transcription factor 1 (USF1), IKAROS family zinc finger 1 (IKZF1), and upstream transcription factor 2, c-fos interacting (USF2) (Additional file 12). This indicates that, even though the cell cycle genes are over-expressed, they are less strongly targeted by these TFs in LCLs compared to blood. To confirm this, we used a permutation analysis using random gene sets equal in size to the cell cycle gene set. Based on this analysis all the negative correlations for these eight TFs were identified as statistically significant (FDR < 0.05).

This analysis suggests that these TFs play a role as negative regulators of the cell cycle. Indeed, many of these TFs are known regulators of the cell cycle, and many have documented roles in repressing genes that promote the cell cycle. For example, SMAD5 can repress transcription, leading to proliferation inhibition after TGF-β signaling [35], E2F8 directly binds to E2F family target genes and repress their transcription [36–38] and ZBTB14 is a transcriptional repressor of the mouse myelocytomatosis oncogene (Myc) gene [39].

To corroborate our network predictions, we examined independent biological evidence to evaluate whether these TFs regulate cell cycle genes. We downloaded the LCL GM12878 ENCODE ChIP-Seq assays for the available TFs (SMAD5, IKZF1, USF1, USF2). We then identified the genes with peaks for these TFs in their promoter region. We also calculated the correlation between the expression of each TF and the cell cycle genes with TF ChIP-Seq binding evidence.

According to the ChIP-Seq data, SMAD5, the TF with the highest inverse correlation between the expression and targeting of cell cycle genes (Fig. 4a), binds to the promoters of 55% of the genes included in our network models. SMAD5 binds to the promoters of cell cycle genes in a much higher proportion; 113 out of the 121 cell cycle genes are bound by SMAD5 (93%). As expected, we found a higher negative correlation between the expression of SMAD5 and the expression of its target genes in cell lines compared to tissue samples in GTEx (*p*-value = 8.7 × 10^−09^, Fig. 4b). The combined GTEx/ENCODE results suggest that cell cycle regulation involves a complex interplay between changes in the expression of regulatory TFs and alterations in the binding of these TFs to their targets.

There is also extensive functional evidence that SMAD5 targets genes to inhibit cellular growth. SMAD proteins have a key role as signal transducers of the TGF-β family members to mediate growth inhibition and apoptosis [40]. SMAD5 negatively regulates cell proliferation during embryonic hematopoiesis [41], in B-cell lymphoma [35], and it induces cell cycle arrest in response to shear stress in tumor cell lines [42].

We repeated this analysis for the three other TFs with ChIP-Seq data available in ENCODE (Additional file 12). Based on the ChIP-Seq data, IKZF1 binds to the promoter region of 20 out of the 121 cell cycle genes, USF1 binds to 52 genes, and USF2 binds to 78 genes. Figure 4c shows a summary visualization of the expression correlation between these four TFs and the cell cycle genes with TF ChIP-Seq binding evidence. The blue edges represent negative correlation between the expression of TFs and their target cell cycle genes, the higher number of blue edges in LCLs compared to blood supports the network-based conclusion that these TFs are negative regulators of cell cycle genes in LCLs. For IKZF1 and USF1 we do not find the same strong negative correlation between the expression of the TFs and their target genes. However, we note that the activity of TFs is not only captured by mRNA levels; and it may also be a result of ligands binding and/or post-translational modification. For example, *USF1* gene regulatory properties depend on its post-translational modification [43]. In contrast to the expression correlation between TFs and target genes, regulatory network analysis may capture the regulatory activity of TFs regardless of differential expression. Experimental analysis at a protein level could confirm the regulatory activity of IKZF1 and USF1.

It has been previously reported that USF1 and USF2 have anti-proliferative roles. For example, loss or impairment of USF transcriptional activity is a common event in cancer cell lines and is associated with increased proliferation [44, 45]. Additionally, over-expression of USF, and in particular USF2, is known to suppress growth in a number of cell lines [46, 47]. Cyclin dependent kinase 4 (CDK4), which controls the progression of cells through G1, is transcriptionally regulated by USF1, USF2 and MYC in non-tumorigenic mammary cells [48]. However, *CDK4* gene regulation and its ability to respond to signals change in breast cancer cell lines, in which USF is transcriptionally inactive and *CDK4* expression regulation independent of both USF and MYC [48]. We found similar differences in *CDK4* regulation for the LCL-vs-blood networks comparison. Consistent with *CDK4* higher expression in LCLs (log_2_ fold change of 2.8, FDR < 0.05), *CDK4* is also less strongly targeted by USF1, USF2, and MYC (edge weight differences of 0.85, 0.82, 0.98, respectively).

For fibroblast-vs-skin comparison, we did not find the same strong negative correlation between cell cycle gene expression and specific TF targeting (Additional file 13). This may be due to the smaller changes we observed in expression of cell cycle genes in fibroblast-vs-skin, in contrast to the LCL-vs-blood comparison. Also, as seen in Fig. 1c, genes in the cell cycle pathway are more expressed in LCLs compared to fibroblasts, which is potentially related to the fact that LCL is a transformed cell line while fibroblast is a primary cell line. However, when we analyzed the relationship between SMAD5 and the cell cycle genes’ expression, we found similar results for fibroblasts (Additional file 13), indicating that some of the patterns we observe for LCLs may be true at a smaller amplitude in other cell line models.

## Discussion

Cell lines are widely used as experimental models to explore basic cellular biology, to study gene regulation, test drug effectiveness and the impact of other compounds on various tissues. One important question is whether cell lines reflect the regulatory processes of the primary tissues from which they are derived. By studying gene expression and gene regulatory networks, we were able to uncover patterns of transcriptional regulation that differentiate cell lines from their tissues of origin. While previous studies focused only on differential expression analysis in a small number of samples, here we used a large set of matched samples to model gene regulatory networks. We were able not only to find the differences in the expression profile of cell lines and their tissues of origin, but also differences in TF regulation at a genome-wide scale. To the best of our knowledge, this is the first study that compares the differences in regulatory networks between cell lines and their tissues of origin, revealing differences in regulatory mechanisms not observed in differential expression analyses.

In comparing LCL-vs-blood and fibroblast-vs-skin, we find that these cell lines and their tissues of origin have important transcriptional differences with approximately 26% of genes being differentially expressed. We identified the drivers of these transcriptional changes by modeling gene regulatory networks and comparing the regulatory networks of cell lines and their tissues of origin. We found that TFs involved in cellular responses to stress and DNA damage, and in the control of cellular growth had the largest changes in targeting. These networks captured the regulatory “rewiring” of TFs and their target genes at a genome-wide scale, and revealed that TFs alter their patterns of regulation in cell lines either through changing their expression or altering the genes they target.

By investigating differential targeting of specific biological processes, we found the most striking difference in the regulation of processes associated with cellular proliferation. Processes including cell cycle, DNA repair, and DNA replication were more highly expressed in cell lines, where they had lower overall targeting by a number of cell cycle-associated TFs that are known to function as repressors. The top TFs found to negatively regulate the expression of cell cycle genes (SMAD5, IKZF1, USF1, USF2) have been previously shown to have a role as transcriptional repressors and as inhibitors of cellular proliferation in tumor cell lines [35, 42, 44, 48]. We validated the negative correlation between the TFs and cell cycle target genes expression using ENCODE Chip-Seq as an independent data set. Our results indicate that cell lines switch off a number of transcriptional repressors, resulting in an overall increase in cell cycle-related transcription.

Many regulatory mechanisms could be mediating these changes including epigenetic changes. For example, a recent study showed hypo-methylation of 250 genes after EBV transformation; in this case the cellular machinery could not maintain DNA methylation [49]. While alterations in the epigenetic profiles of LCLs have been demonstrated [22, 50], our analysis is the first to explore the changes of TFs regulatory targeting. Studying tissue heterogeneity and cell type-specific characteristics could also reveal important regulatory mechanisms that differentiate cell lines from tissues. Studies comparing bulk RNA-Seq to single cell RNA-Seq have shown the power of single cell RNA-Seq approaches to uncover tissue heterogeneity, and also how computational deconvolution approaches can be used to measure the cell type composition of mixed tissues [3, 51–54].

The fact that the cell lines in the GTEx data set were created in very different ways – one transformed and the other a primary cell line – suggests that the global alterations we observe in both types of cell lines in terms of transcriptional patterns may be associated with growing in culture, the lack of tissue context, and decreased cellular heterogeneity. Some of the changes may also be associated with the transformation process as we observed smaller changes in expression and regulation of cell cycle genes in fibroblasts compared to LCLs.

Our regulatory network analysis captured differences in the cell line regulatory processes that could not have been captured using more standard approaches such as differential expression analysis. For example, we identified TFs, such as SP1 and SP3, that were not differentially expressed between cell lines and their tissues of origin, but targeted different genes. Additionally, we identified the transcriptional regulatory differences between the cell lines and tissues that are associated with the cell cycle genes’ differential expression. Our analysis focused on regulation via promoters and did not include long-range enhancer regulation. While many commonly used network methods are based on pairwise co-expression information that does not fully capture regulatory processes [55–57], PANDA’s message-passing approach aims to infer complex regulatory relationships between TFs and their target genes. PANDA also has the advantage of integrating different types of genomic data to give more informative results, and it outperforms other network methods in its ability to predict TF binding site occupancy validated by ChIP data [28].

## Conclusions

In our analysis we found that biological processes are differentially targeted by TFs in LCLs and fibroblast cell lines compared to their tissues of origin. While the existing literature includes evidence that cell lines have a higher expression of genes associated with proliferation [21, 22], here we were able to identify the key transcriptional process that drives these differences by applying regulatory network analysis. We were able to specifically find a number of cell cycle-associated TFs that are known to function as repressors that are less strongly regulating cell cycle genes in cell lines compared to their tissues of origin.

Understanding that differences exist between cell lines and tissues in patterns of TF targeting is important for designing and interpreting experimental studies using cell line models. This is especially true for the development of targeted therapeutics, where targeted pathways may be altered in cell lines relative to the tissues from which they are derived. Our analysis demonstrates that cell lines exhibit both gene expression and regulatory changes that distinguish them from their primary tissues, provides insights into which transcriptional processes are altered, and identifies several regulators that are likely mediating those changes. In addition to considering gene expression changes, considering regulatory network topologies allows for a more complete understanding of the regulatory differences between cell lines and their tissues of origin.

## Methods

### GTEx data

The GTEx version 6.0 RNA-Seq data set (phs000424.v6.p1, 2015–10-05 released) was downloaded from dbGaP (approved protocol #9112). Using YARN R package (version 1.0.0) we performed quality control, gene filtering, and normalization preprocessing [58]. We grouped related body regions using gene expression similarity. For example, skin samples from the lower leg (sun exposed) and from the suprapubic region (sun unexposed) were grouped as “skin.” We filtered and normalized the data in a tissue-aware manner using smooth quantile normalization [github.com/stephaniehicks/qsmooth] [59]. The final data set contains 549 research subjects (188 females and 361 males) comprising 38 tissues (which included two cell lines), 30,333 genes, and 9435 samples. We filtered sex-chromosome and mitochondrial genes (retaining 29,242 genes).

We reduced the data set to only cell line and tissue-specific paired samples, which comprised 127 subjects with whole blood and LCL samples, and 244 subjects with skin and primary fibroblast cell line samples; 89 subjects have data across all four groups. For the skin samples, an equivalent number of samples were obtained from the lower leg (*n* = 123), and from the suprapubic region (*n* = 121). We kept only the 27,175 genes with at least one TF binding motif in its promoter region (see section: Gene regulatory networks), so that we could use the same set of genes for differential expression and gene regulatory network analysis.

GTEx version 6.0 RNA-Seq was annotated using the GENCODE release 19 (GRCh37.p13). Thus, we defined the different types of genes (protein coding, antisense, pseudogene, lincRNA, and other) according to the same genome annotation downloaded from http://www.gencodegenes.org/releases/19.html.

### Principal components analysis

We performed principal component analysis (PCA) as implemented in the plotOrd function on the R package metagenomeSeq 1.12.1. PCA was applied to the full expression data matrix.

### Differential expression analysis

Differential expression analysis was performed using voom available in the limma Bioconductor R package (version 3.26.9) [24]. We performed four analyses using only paired samples between the groups of comparison: 1) LCL (*n* = 127) and Blood (*n* = 127), 2) Fibroblast (*n* = 244) and Skin (*n* = 244), 3) LCL (*n* = 89) and Fibroblast (*n* = 89), 4) Blood (*n* = 89) and Skin (*n* = 89). Multiple testing correction was performed using Benjamini-Hochberg. Genes with adjusted *p*-values less than an alpha of 0.05 and an absolute log_2_ fold change greater than 2 were considered differentially expressed.

### Pathway enrichment analysis

We performed GSEA to determine the biological functions related to the differential expression between cell lines and tissues [25]. All genes were ranked by the moderated *t*-statistic produced by voom differential expression analysis. We used pre-ranked GSEA program (Java command line version 2–2.0.13) to calculate a running-sum statistic. We used the gene sets obtained from the KEGG pathway database that was downloaded from the Molecular Signatures Database (MSigDB) (http://www.broadinstitute.org/gsea/msigdb/collections.jsp) (“c2.cp.kegg.v5.0.symbols.gmt”). We performed 1000 gene set permutations to assess the statistical significance, and considered gene sets with FDR < 0.05 significant. We only considered gene sets of size greater than 15 and less than 500 genes after filtering out those genes not in the expression data set, or 176 gene sets in total.

### Gene regulatory networks

We reconstructed gene regulatory networks using PANDA, a message-passing model that integrates multiple types of genomic data and infers the network of interactions between TFs and their target genes [28]. PANDA starts with a prior regulatory network inferred by mapping TF binding sites to the genome, integrates PPI and gene expression data to iteratively refine the network structure and deduces a final consensus regulatory network. In the regulatory networks estimated by PANDA, each edge connects a TF to a target gene, and the edge weight indicates the strength of the inferred regulatory relationship.

PANDA also iteratively refines other two network types: the cooperativity network, which captures synergistic interactions between TFs (initially estimated with PPI data), and the co-regulatory network, which captures co-regulatory patterns between genes (initially estimated with gene co-expression data). However, we limited our analysis to the regulatory network.

We generated one PANDA regulatory network for each group: LCL, blood, fibroblast, and skin (Additional file 6) [28]. For each network, we used the same TF/target gene prior regulatory network and the same PPI prior network (see below). In creating the gene regulatory network models, we used PANDA’s default parameters: the model was run until it achieved a hamming distance of 0.001 and the update parameter (alpha) was 0.1.

To generate the TF/target gene regulatory prior, we downloaded all position weight matrices (PWM) for direct and inferred *Homo sapiens* motifs from the Catalog of Inferred Sequence Binding Preferences (CIS-BP) (2015–07-07) [60]. For each TF, we selected the motif with the highest information content, total of 695 motifs. We mapped the PWMs for these 695 motifs to promoter regions of Ensembl gene (ENSG) ids using FIMO [61]. Motif mappings were parsed to only retain those below *p*-value cut-off of 10^−5^ and ranging from -750 bp to +250 bp around the transcription start site (TSS). A p-value cut-off of 10^−5^ was chosen to balance the accuracy of TF/target gene predictions and the density of the corresponding network of interactions (for a p-value accuracy below 10^−5^, the regulatory prior had approximately 9% of TF and target gene interactions). Finally, we kept only TFs with at least one significant promoter hit and genes that were found expressed in the GTEx filtered and normalized data set, which resulted in a TF/target gene prior of 652 TFs and 27,175 target genes.

To generate the PPI prior, we downloaded *Homo sapiens* PPI interactions (9606.protein.links.v10.txt.gz) and protein aliases (9606.protein.aliases.v10.txt.gz) from StringDb v10 (2015–10-27). We parsed this PPI data set for the 652 TFs in our TF/target gene prior. To make the PPI prior in the same scale as the regulatory prior, the PPI interaction scores were divided by 1000 (making its range 0 to 1); self- interactions were set equal to one.

To run PANDA, for each sample group, we used the TF/target gene prior, the PPI prior, and the sample group gene expression data. The TF/target gene edge weights emerging from PANDA were then used to compare networks between each cell line and its tissue of origin. For pairs of networks, we compared the TF out-degree, defined as the sum of edge weights from that TF, and the gene in-degree, defined as the sum of all incoming edge weights a gene received from all expressed TFs in the network. The illustrations of the subnetworks were done using Cytoscape default yFiles Organic layout (version 3.4.0) [62] where each edge connects a TF to a target gene, and the edge weight is represented by the color shade.

To assess the robustness of the regulatory network models, we used bootstrap sub-sampling of the RNA-Seq datasets from the 89 paired samples across all four groups. We did multiple random selections of 40 paired samples, and generated 100 networks for each group: LCL, blood, fibroblast, and skin. We then calculated the average and standard deviation of the edge weights across the bootstrapped networks and compared to the network obtained from all the samples. We also compared degree centrality measures (more specifically, the weighted out-degree and in-degree differences between the corresponding cell line and tissue networks) estimated from the bootstrapped networks and the network obtained using all the samples.

### ENCODE Chip-Seq data

Chip-Seq on GM12878 (type of LCL) targeting the TFs SMAD5, IKZF1, USF1, and USF2 were downloaded from the ENCODE Project (https://www.encodeproject.org, accessed 2016–06-03). We used the narrow peak data processed by ENCODE from 2 biological replicates (accession: ENCFF553HHF, ENCFF001VEJ, ENCFF002CIB, ENCFF001VFQ). Then, to identify genes bound by each of these TFs we used *bedtools* (v2.17) to annotate peaks that fall within the promoter region of a gene (same promoter regions used for the network reconstruction, ranging from -750 bp to +250 bp of the TSS).

## Additional files

Additional file 1: Similarity between cell lines and their tissues of origin based on gene expression. (A) Principal component analysis (PCA) was performed to evaluate possible batch effects in the gene expression data. Samples are labeled based on the year the sample was analyzed by the GTEx project, and the plots show the sample separation for the first 7 PCs. (B) Number of genes expressed in each group (LCL, whole blood, fibroblast, skin). Genes were separated into biological classes using the definitions from GENCODE release 19 (GRCh37.p13). (C) PCA of paired samples between the two tissues and cell lines (total of 89 subjects with all four samples) based on the normalized expression of all genes. The primary axis separates samples by tissue; the secondary axis separates primary tissue from cell lines. (D) To access whether the PCA results were dependent on the 89 samples chosen because they were present in all four groups, we repeated the analysis 100 times using 89 randomly selected samples in each group. The left panel shows the projection of the first 2 PCs for one random analysis, and right panel shows the distribution of PC1 and PC2 for each of the 100 analyses. (PDF 267 kb)

Additional file 2: Gene expression variability. (A) Density plot of the gene expression standard deviation (SD) within each cell line/tissue group. (B) F-test was performed to evaluate the differences in gene expression variance between the indicated groups. The histograms show the ratio of variances at log scale for all the genes, and the red line indicates similar gene expression variance between the two indicated groups. The bar plots show the percentage of genes with significant differences in variance (FDR < 0.05). (PDF 337 kb)

Additional file 3: Differential expression analysis. (A) Volcano plots of the differential expression analysis using voom on paired samples between the indicated groups. The lines indicate a log_2_ fold change of −2 or 2. (B) Percentage of genes called differentially expressed (DE) varying the log_2_ fold change at a FDR < 0.05. (PDF 1114 kb)

Additional file 4: Differentially expressed genes in each of the comparisons: LCL-vs-blood; fibroblast-vs-skin; blood-vs-skin; LCL-vs-fibroblast (absolute log_2_ fold change >2 and FDR < 0.05). (XLSX 2130 kb)

Additional file 5: Pathway enrichment analysis significance performed by GSEA. (XLSX 90 kb)

Additional file 6: Reconstruction and robustness of gene regulatory networks. (A) A cartoon of how the networks were generated. We used PANDA, a message-passing network inference algorithm that integrates multiple types of genomic data and infers the network of interactions between TFs and their target genes. PANDA uses a prior regulatory network inferred by mapping TF binding sites to the genome (motif data), integrates protein-protein interaction data and group-specific gene expression data to iteratively refine and deduce a final regulatory network. We generated one PANDA network for each group: LCL, whole blood, fibroblasts, and skin. The illustrations represent an example subnetwork with 5 TFs and 50 of its target genes. The strength of the inferred regulatory relationship is indicated by the edge thickness. Next, we did multiple random selections of 40 paired samples, and generated 100 networks for each group: LCL, blood, fibroblast, and skin. (B) Density plot of the standard deviation of the edge weights across the 100 bootstrapped networks in each group: LCL, blood, fibroblast, and skin. (C) Scatter plot of the average edge weights obtained from the bootstrapped networks and the edge weights from the network obtained using all the samples. (D) Scatter plot of the TF out-degree differences between the indicated cell line and tissue for the bootstrapped networks versus the network obtained using all the samples. (E) Scatter plot of the gene in-degree differences between the indicated cell line and tissue for the bootstrapped networks versus the network obtained using all the samples. (PDF 1025 kb)

Additional file 7: Transcription factors differentially-targeting genes in cell lines and their tissues of origin. (A) Distribution of TF out-degree difference for LCL-vs-blood networks comparison (red) and for fibroblast-vs-skin networks comparison (blue). Positive values indicate higher targeting in cell lines, and negative values indicate higher targeting in tissues. (B) Scatter plots of *t*-statistic values for TF differential expression (voom) and “differential targeting” (paired t-test to compare the TF out-going edge weights between the cell line and tissue-specific networks) comparing LCL versus blood (left panel); and fibroblasts versus skin (right panel). Red: TFs that achieved significance for differential expression (FDR < 0.05 and absolute log_2_ fold change >2) and for differential targeting (FDR < 0.05). (PDF 82 kb)

Additional file 8: Function of the TFs with the largest difference in out-degree comparing LCL-vs-blood; and fibroblast-vs-skin regulatory networks. (XLSX 41 kb)

Additional file 9: Significance of the in-degree difference of genes belonging to a specific pathway against genes not in the pathway using an unpaired *t*-test. (XLSX 36 kb)

Additional file 10: Transcriptional targeting of genes in the pathways over-expressed for both cell lines. Boxplot of the gene in-degree differences for the genes in the specified pathway and for genes not in the pathway (*FDR < 0.05 *t*-test). Reduction of gene in-degree difference indicates that the genes in the pathway are less targeted by TFs in the cell line compared to its tissue of origin. (PDF 140 kb)

Additional file 11: List of genes in the KEGG cell cycle pathway that are found expressed in our data set. (XLSX 27 kb)

Additional file 12: Transcription factors differentially regulating genes in the cell cycle pathway in LCLs compared to blood. (A) Spearman correlation between the log_2_ fold change in gene expression (LCL-vs-blood comparison) of KEGG cell cycle pathway genes and the differential targeting they receive by the specified TF. Red: evidence of TF ChIP-Seq binding on the promoter of the gene, black: no evidence of TF binding. The negative correlation observed indicates the cell cycle genes are more highly expressed but less targeted by the TF in LCL compared to blood. (B) Boxplot of Spearman correlation coefficients between TF expression levels and expression levels of all genes, and between TF expression levels and the expression levels of cell cycle genes with TF ChIP-Seq binding evidence for LCL and blood samples. Significance is based on a Wilcoxon rank-sum test for LCL-vs-blood comparison. (PDF 213 kb)

Additional file 13: Cell cycle genes regulation by SMAD5 in fibroblast and skin samples. (A) Spearman correlation between the log_2_ fold change in gene expression (fibroblast-vs-skin comparison) of KEGG cell cycle pathway genes and the differential targeting they receive by the TF SMAD5. Blue: evidence of SMAD5 ChIP-Seq binding, black: no evidence of SMAD5 binding. (B) Boxplot of Spearman correlation coefficients between SMAD5 expression levels and expression levels of all genes, and between SMAD5 expression levels and the expression levels of cell cycle target genes with SMAD5 ChIP-Seq binding evidence for fibroblast and skin samples. Significance is based on a Wilcoxon rank-sum test for fibroblast-vs-skin comparison. (PDF 79 kb)
