# Supplementary figures and images for: Regulatory network changes between cell lines and their tissues of origin
Source: BMC Genomics. 2017 Sep 12;18:723. doi: 10.1186/s12864-017-4111-x (PMC5596945; doi:10.1186/s12864-017-4111-x)

A

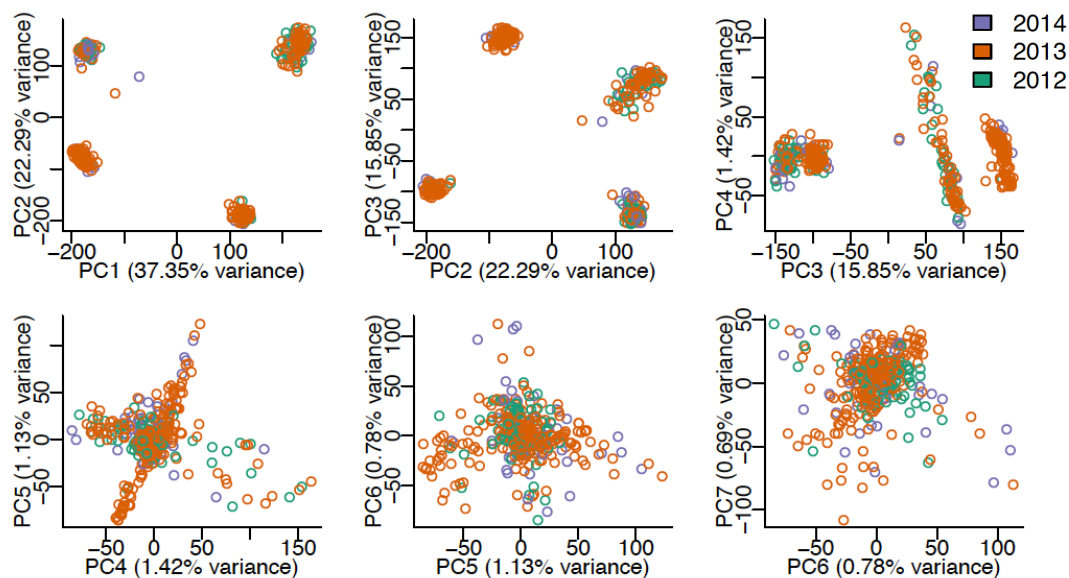

B

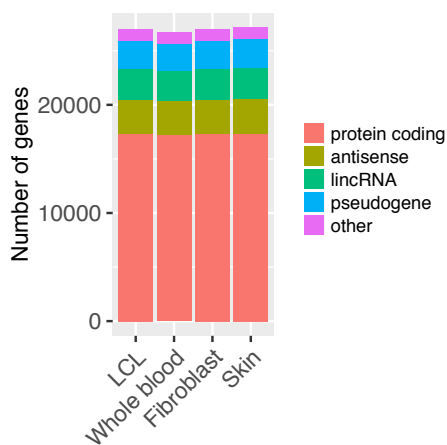

C

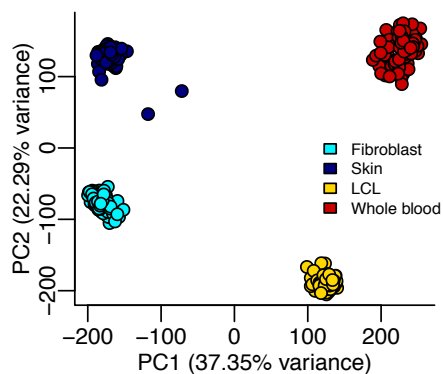

D

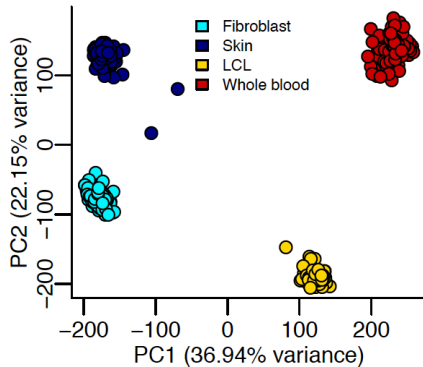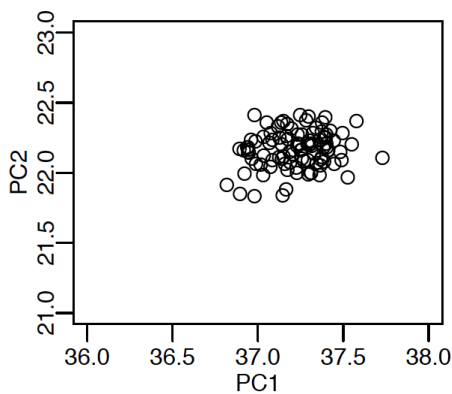

Supplement: Supplementary file 1 — Similarity between cell lines and their tissues of origin based on gene expression. (A) Principal component analysis (PCA) was performed to evaluate possible batch effects in the gene expression data. Samples are labeled based on the year the sample was analyzed by the GTEx project, and the plots show the sample separation for the first 7 PCs. (B) Number of genes expressed in each group (LCL, whole blood, fibroblast, skin). Genes were separated into biological classes using the definitions from GENCODE release 19 (GRCh37.p13). (C) PCA of paired samples between the two tissues and cell lines (total of 89 subjects with all four samples) based on the normalized expression of all genes. The primary axis separates samples by tissue; the secondary axis separates primary tissue from cell lines. (D) To access whether the PCA results were dependent on the 89 samples chosen because they were present in all four groups, we repeated the analysis 100 times using 89 randomly selected samples in each group. The left panel shows the projection of the first 2 PCs for one random analysis, and right panel shows the distribution of PC1 and PC2 for each of the 100 analyses. (PDF 267 kb) [file 12864_2017_4111_MOESM1_ESM.pdf]

A

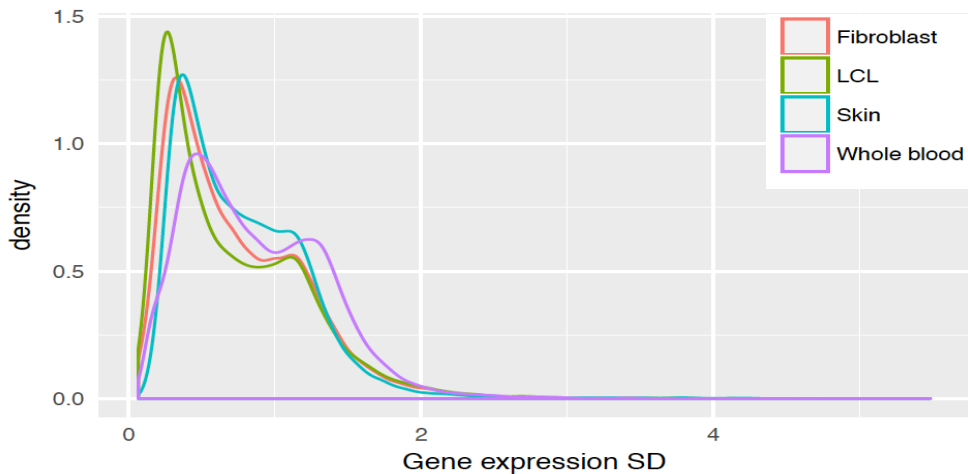

B

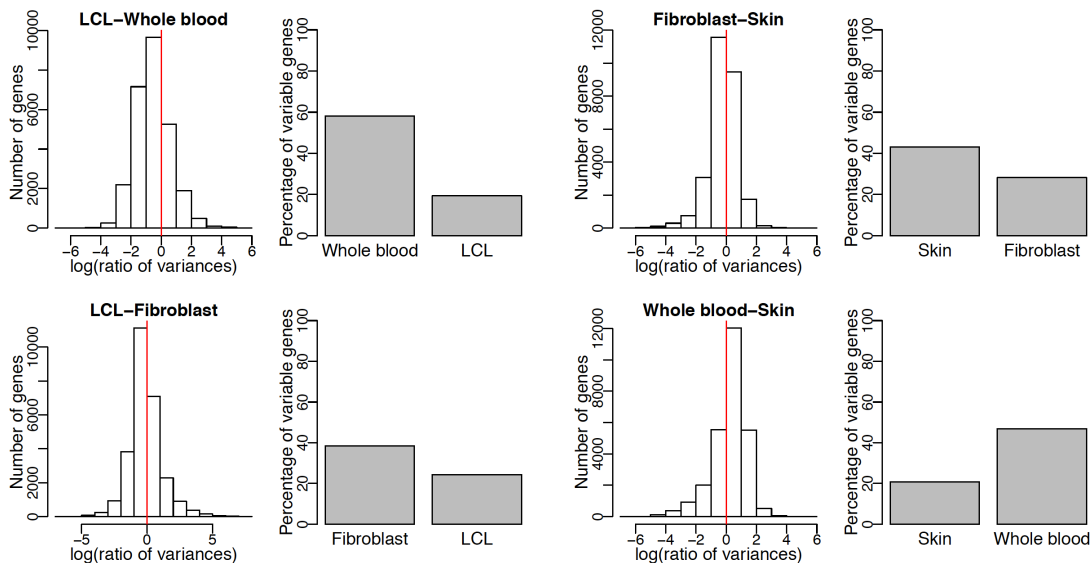

Supplement: Supplementary file 2 — Gene expression variability. (A) Density plot of the gene expression standard deviation (SD) within each cell line/tissue group. (B) F-test was performed to evaluate the differences in gene expression variance between the indicated groups. The histograms show the ratio of variances at log scale for all the genes, and the red line indicates similar gene expression variance between the two indicated groups. The bar plots show the percentage of genes with significant differences in variance (FDR < 0.05). (PDF 337 kb) [file 12864_2017_4111_MOESM2_ESM.pdf]

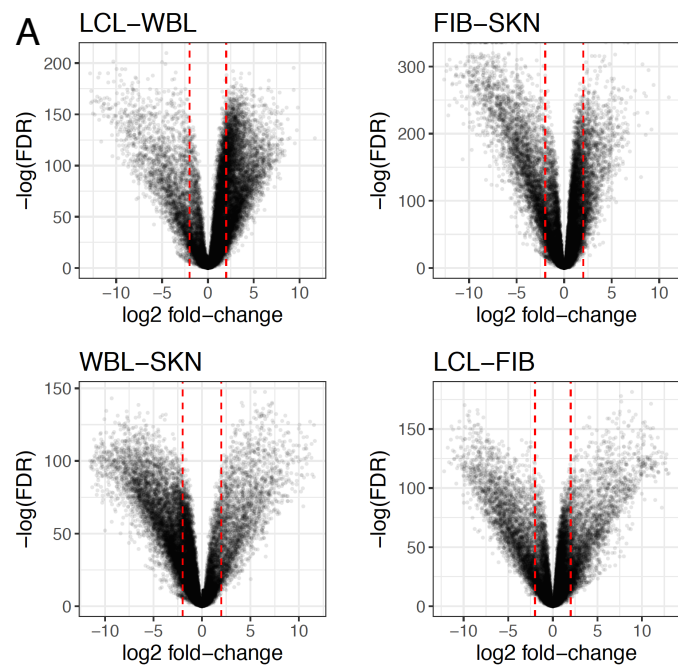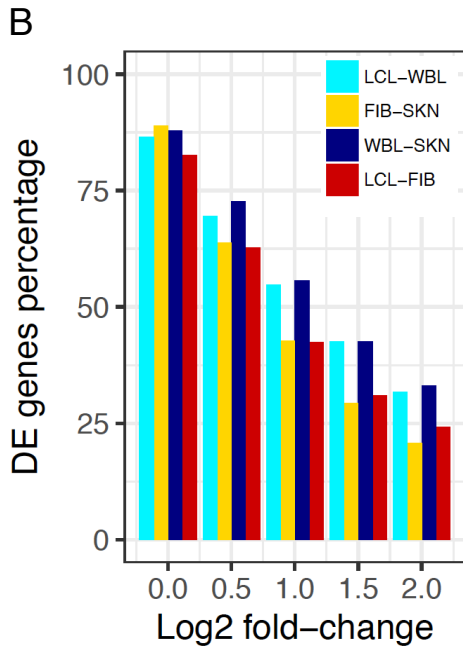

Supplement: Supplementary file 3 — Differential expression analysis. (A) Volcano plots of the differential expression analysis using voom on paired samples between the indicated groups. The lines indicate a log2 fold change of −2 or 2. (B) Percentage of genes called differentially expressed (DE) varying the log2 fold change at a FDR < 0.05. (PDF 1114 kb) [file 12864_2017_4111_MOESM3_ESM.pdf]

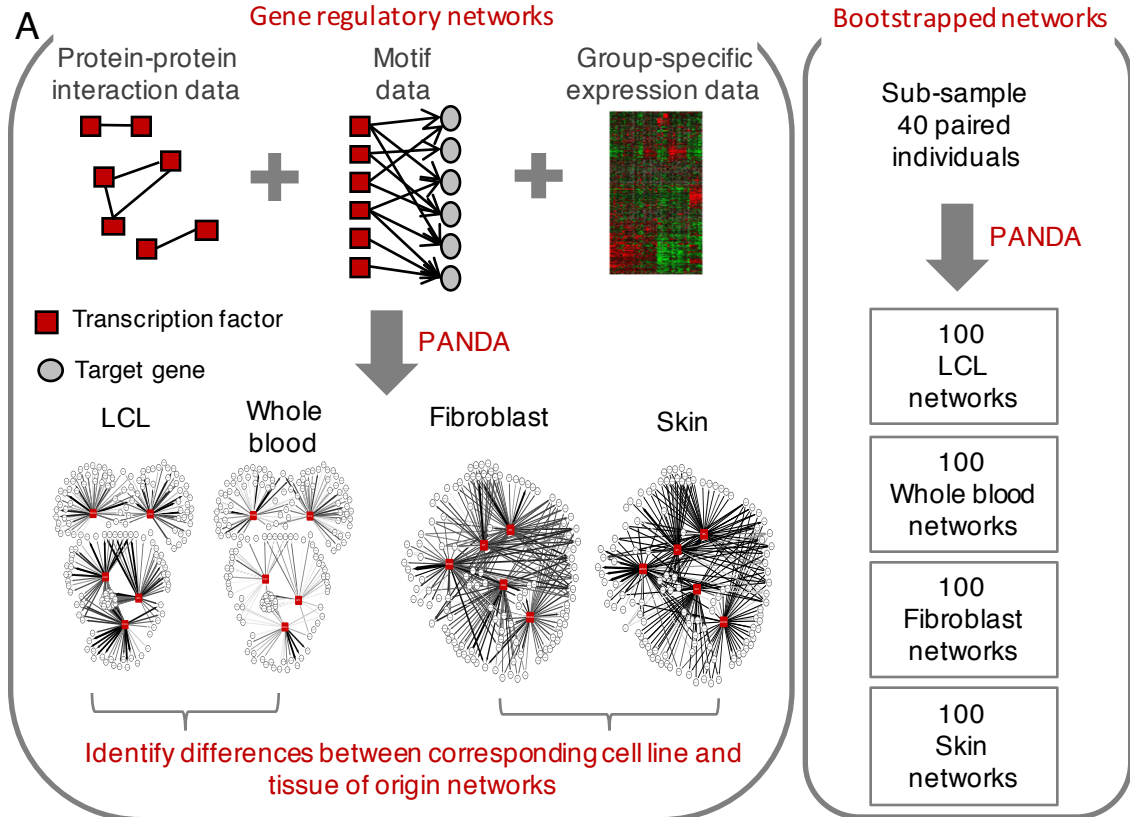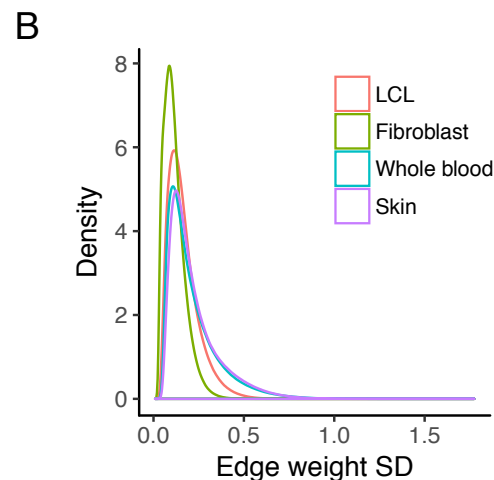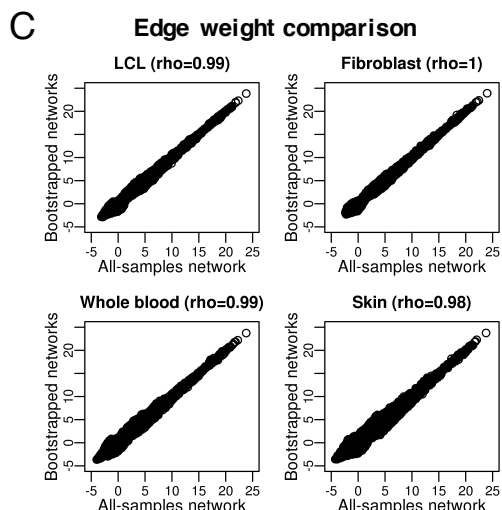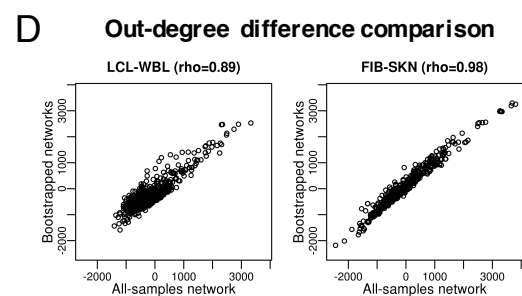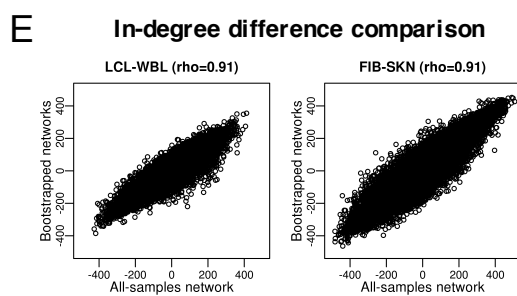

Supplement: Supplementary file 6 — Reconstruction and robustness of gene regulatory networks. (A) A cartoon of how the networks were generated. We used PANDA, a message-passing network inference algorithm that integrates multiple types of genomic data and infers the network of interactions between TFs and their target genes. PANDA uses a prior regulatory network inferred by mapping TF binding sites to the genome (motif data), integrates protein-protein interaction data and group-specific gene expression data to iteratively refine and deduce a final regulatory network. We generated one PANDA network for each group: LCL, whole blood, fibroblasts, and skin. The illustrations represent an example subnetwork with 5 TFs and 50 of its target genes. The strength of the inferred regulatory relationship is indicated by the edge thickness. Next, we did multiple random selections of 40 paired samples, and generated 100 networks for each group: LCL, blood, fibroblast, and skin. (B) Density plot of the standard deviation of the edge weights across the 100 bootstrapped networks in each group: LCL, blood, fibroblast, and skin. (C) Scatter plot of the average edge weights obtained from the bootstrapped networks and the edge weights from the network obtained using all the samples. (D) Scatter plot of the TF out-degree differences between the indicated cell line and tissue for the bootstrapped networks versus the network obtained using all the samples. (E) Scatter plot of the gene in-degree differences between the indicated cell line and tissue for the bootstrapped networks versus the network obtained using all the samples. (PDF 1025 kb) [file 12864_2017_4111_MOESM6_ESM.pdf]

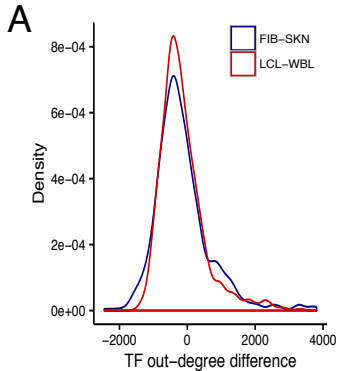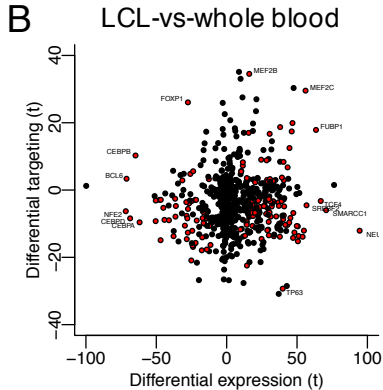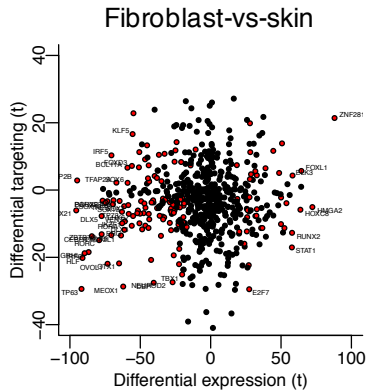

Supplement: Supplementary file 7 — Transcription factors differentially-targeting genes in cell lines and their tissues of origin. (A) Distribution of TF out-degree difference for LCL-vs-blood networks comparison (red) and for fibroblast-vs-skin networks comparison (blue). Positive values indicate higher targeting in cell lines, and negative values indicate higher targeting in tissues. (B) Scatter plots of t-statistic values for TF differential expression (voom) and “differential targeting” (paired t-test to compare the TF out-going edge weights between the cell line and tissue-specific networks) comparing LCL versus blood (left panel); and fibroblasts versus skin (right panel). Red: TFs that achieved significance for differential expression (FDR < 0.05 and absolute log2 fold change >2) and for differential targeting (FDR < 0.05). (PDF 82 kb) [file 12864_2017_4111_MOESM7_ESM.pdf]

Gene in-degree difference

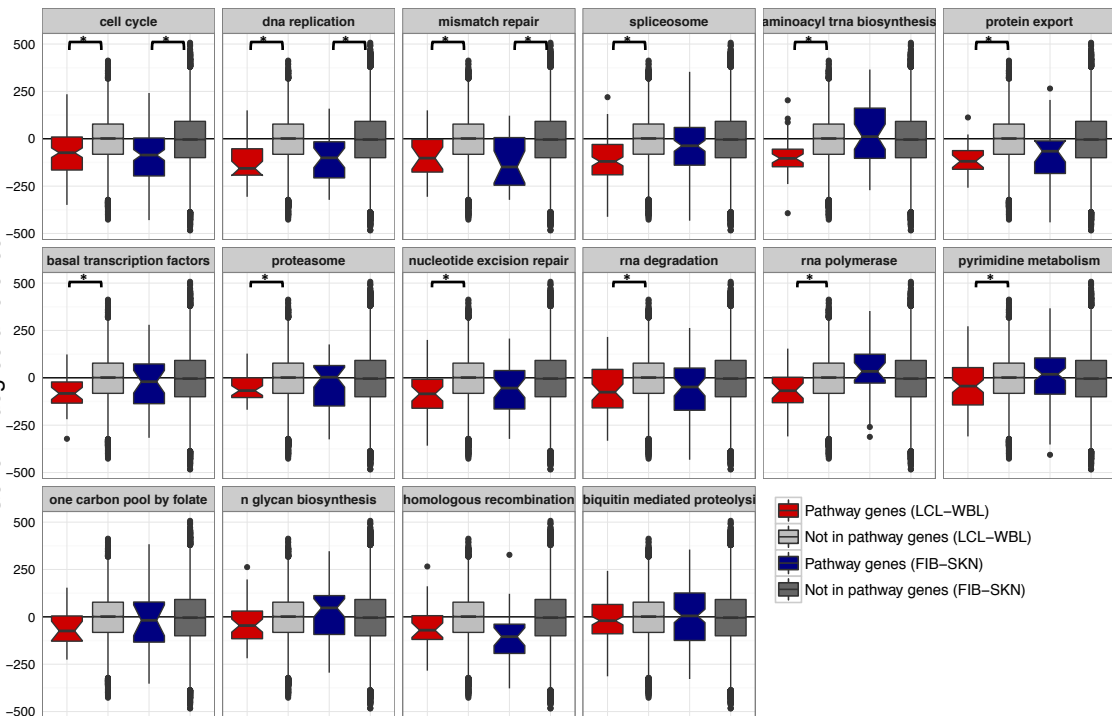

Supplement: Supplementary file 10 — Transcriptional targeting of genes in the pathways over-expressed for both cell lines. Boxplot of the gene in-degree differences for the genes in the specified pathway and for genes not in the pathway (*FDR < 0.05 t-test). Reduction of gene in-degree difference indicates that the genes in the pathway are less targeted by TFs in the cell line compared to its tissue of origin. (PDF 140 kb) [file 12864_2017_4111_MOESM10_ESM.pdf]

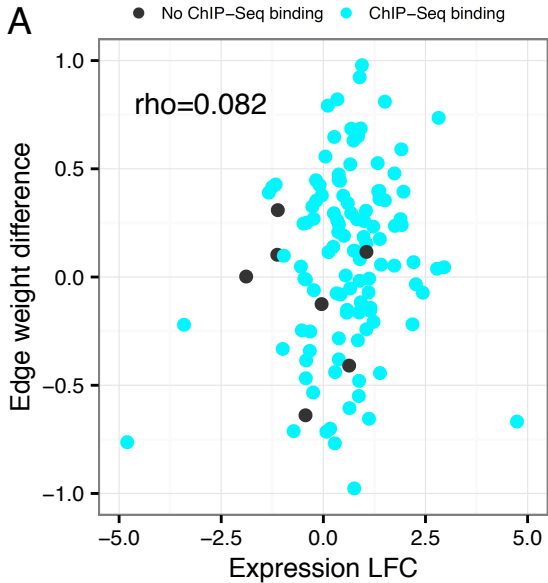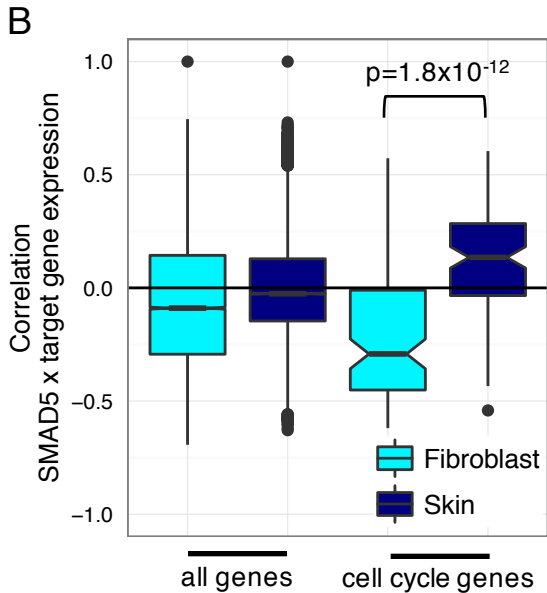

Supplement: Supplementary file 13 — Cell cycle genes regulation by SMAD5 in fibroblast and skin samples. (A) Spearman correlation between the log2 fold change in gene expression (fibroblast-vs-skin comparison) of KEGG cell cycle pathway genes and the differential targeting they receive by the TF SMAD5. Blue: evidence of SMAD5 ChIP-Seq binding, black: no evidence of SMAD5 binding. (B) Boxplot of Spearman correlation coefficients between SMAD5 expression levels and expression levels of all genes, and between SMAD5 expression levels and the expression levels of cell cycle target genes with SMAD5 ChIP-Seq binding evidence for fibroblast and skin samples. Significance is based on a Wilcoxon rank-sum test for fibroblast-vs-skin comparison. (PDF 79 kb) [file 12864_2017_4111_MOESM13_ESM.pdf]
