# Supplementary material for: Regulatory network changes between cell lines and their tissues of origin
Source: BMC Genomics. 2017 Sep 12;18:723. doi: 10.1186/s12864-017-4111-x (PMC5596945; doi:10.1186/s12864-017-4111-x)
Supplement: Supplementary file 12 — Transcription factors differentially regulating genes in the cell cycle pathway in LCLs compared to blood. (A) Spearman correlation between the log2 fold change in gene expression (LCL-vs-blood comparison) of KEGG cell cycle pathway genes and the differential targeting they receive by the specified TF. Red: evidence of TF ChIP-Seq binding on the promoter of the gene, black: no evidence of TF binding. The negative correlation observed indicates the cell cycle genes are more highly expressed but less targeted by the TF in LCL compared to blood. (B) Boxplot of Spearman correlation coefficients between TF expression levels and expression levels of all genes, and between TF expression levels and the expression levels of cell cycle genes with TF ChIP-Seq binding evidence for LCL and blood samples. Significance is based on a Wilcoxon rank-sum test for LCL-vs-blood comparison. (PDF 213 kb) [file 12864_2017_4111_MOESM12_ESM.pdf]

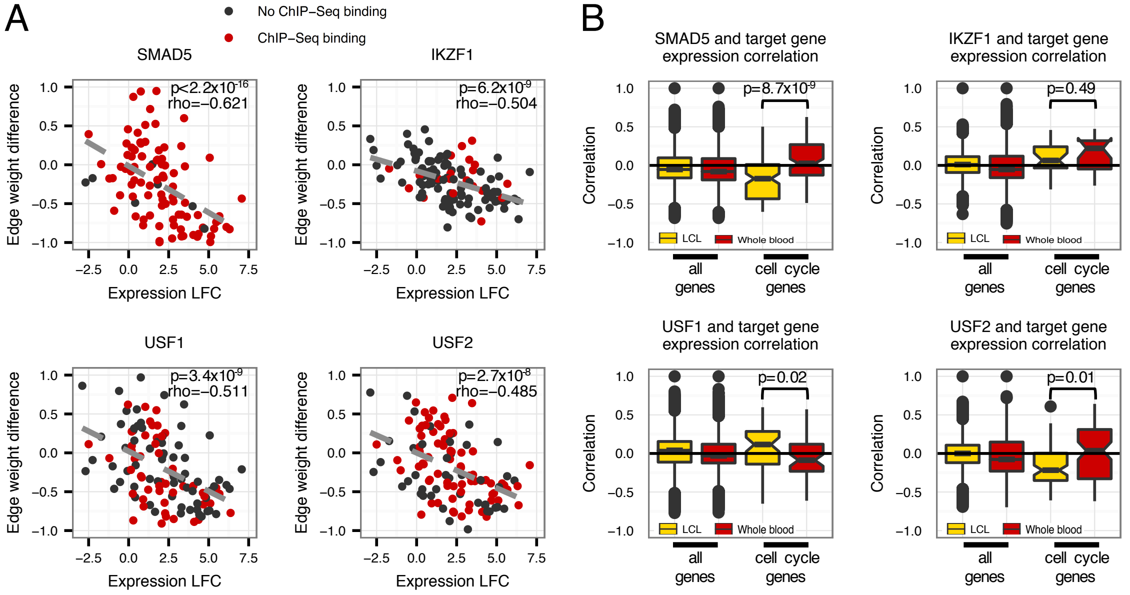

A
No ChIP−Seq binding
ChIP−Seq binding
SMAD5
p<2.2x10-16
rho=−0.621
IKZF1
p=6.2x10-9
rho=−0.504
USF1
p=3.4x10-9
rho=−0.511
USF2
p=2.7x10-8
rho=−0.485
Edge weight difference
Expression LFC
B
SMAD5 and target gene expression correlation
p=8.7x10-9
IKZF1 and target gene expression correlation
p=0.49
USF1 and target gene expression correlation
p=0.02
USF2 and target gene expression correlation
p=0.01
Correlation
LCL
Whole blood
all genes
cell cycle genes
